# Assessing Saccadic Eye Movements With Head-Mounted Display Virtual Reality Technology

**DOI:** 10.3389/fpsyt.2020.572938

**Published:** 2020-09-17

**Authors:** Yu Imaoka, Andri Flury, Eling D. de Bruin

**Affiliations:** ^1^ Motor Control & Learning Laboratory, Institute of Human Movement Sciences and Sport, Department of Health Sciences and Technology, ETH Zurich, Zurich, Switzerland; ^2^ Division of Physiotherapy, Department of Neurobiology, Care Sciences and Society, Karolinska Institute, Stockholm, Sweden

**Keywords:** saccadic eye movement, virtual reality, head mounted display (HMD), dementia, neurological disorder, ageing, pupillary response, saccade

## Abstract

As our society is ageing globally, neurodegenerative disorders are becoming a relevant issue. Assessment of saccadic eye movement could provide objective values to help to understand the symptoms of disorders. HTC Corporation launched a new virtual reality (VR) headset, VIVE Pro Eye, implementing an infrared-based eye tracking technique together with VR technology. The purpose of this study is to evaluate whether the device can be used as an assessment tool of saccadic eye movement and to investigate the technical features of eye tracking. We developed a measurement system of saccadic eye movement with a simple VR environment on Unity VR design platform, following an internationally proposed standard saccade measurement protocol. We then measured the saccadic eye movement of seven healthy young adults to analyze the oculo-metrics of latency, peak velocity, and error rate of pro- and anti-saccade tasks: 120 trials in each task. We calculated these parameters based on the saccade detection algorithm that we have developed following previous studies. Consequently, our results revealed latency of 220.40 ± 43.16 ms, peak velocity of 357.90 ± 111.99°*/*s, and error rate of 0.24 ± 0.41% for the pro-saccade task, and latency of 343.35 ± 76.42 ms, peak velocity of 318.79 ± 116.69°*/*s, and error rate of 0.66 ± 0.76% for the anti-saccade task. In addition, we observed pupil diameter of 4.30 ± 1.15 mm (left eye) and 4.29 ± 1.08 mm (right eye) for the pro-saccade task, and of 4.21 ± 1.04 mm (left eye) and 4.22 ± 0.97 mm (right eye) for the anti-saccade task. Comparing between the descriptive statistics of previous studies and our results suggests that VIVE Pro Eye can function as an assessment tool of saccadic eye movement since our results are in the range of or close to the results of previous studies. Nonetheless, we found technical limitations especially about time-related measurement parameters. Further improvements in software and hardware of the device and measurement protocol, and more measurements with diverse age-groups and people with different health conditions are warranted to enhance the whole assessment system of saccadic eye movement.

## Introduction

Our society is ageing worldwide. According to the United Nations (UN), the number of people aged 65 years and over is projected to increase from 0.7 billion (9% of the global population) in 2019 to 1.5 billion (16%) in 2050 (1). In addition, the elderly people aged 60 years and over are expected to outnumber the children under 5 years in 2020 for the first time in our history (2). Because of this development, the World Health Organization (WHO) launched The Decade of Healthy Ageing (2020–2030), an opportunity to bring together relevant stakeholders to improve the lives of older people in our entire society (2). Particularly, WHO emphasizes the importance of developing guidance and measurement tools for primary care providers so that they can assess health status of the elderly more comprehensively to slow and/or reverse the declines in their physical and mental capacities (3, 4).

Among various relevant diseases and disorders confronting us in the growing ageing society, neurodegenerative diseases are becoming more prevalent (5). It is reported that neurological disorders were the leading cause of Disability-Adjusted Life Year (DALY) in 2015, accounting for 10.2% of global DALYs, and were the second leading cause of deaths, comprising 16.8% of global deaths (6). Detecting early disease-related symptoms would facilitate implementation of preventive measures. In this context, it has been hypothesized that assessment of eye movements can be invaluable for healthcare providers because eye tracking provides indirect access to the neural and cognitive processing in a simple manner (7–9) and associates with neurodegeneration (10). Ocular movements can be subcategorized into two clauses: 1) fixation, blink, vergence, smooth pursuit, vestibuloocular reflexes, optokinetic nystagmus, and pupillary responses, and 2) saccade (7, 9). While it is important to evaluate eye movements with combining these subcategorized events (9), saccadic eye movement is often assessed in various neurodegenerative disorders; e.g., in dementia (11).

A saccade refers to a rapid and conjugate eye movement that voluntarily shifts the eyes from one target to another (12). We usually perform the saccades, initiating the movement within 250 ms, shifting our eyes at the speed up to 700°/s, and completing the shift in 30 ∼ 100 ms (12, 13). Saccades are generally subdivided into two main classes: 1) saccades that are made in response to an external guide of visual stimuli and 2) saccades that are performed without visual target (14). These two types of saccades work differently in terms of the brain processing. One of the frequently used methods for the former type of saccade is the visually guided saccade (VGS). The subjects first look at a central fixed target and then have to move their eyes to another target that appears at some point outside the center of their visual field after the central target disappears. An example paradigm of the latter type of saccade is called the memory-guided saccade (MGS). The saccade procedure is similar to the VGS; however, the subjects need to remember for a short time the location of another target toward which they have to make a saccade. In performing a saccade, several cortical areas play an important role: frontal eye field (FEF), supplementary eye field (SEF), parietal eye field (PEF) or posterior parietal cortex (PPC), and superior colliculus (SC) (14). Specifically, FEF and PPC are important to initiate a saccade. The parietal cortex generally contributes to triggering automatic or reflexive saccades, while the frontal lobe, including the FEF, is affected by more cognitively demanding eye movement tasks. After SC receives the signals from FEF, SEF, and PEF (or PPC), it sends out next signals to the saccade generators in the brainstem. Finally, the brainstem saccade generator sends its outputs to the motoneurons of the oculomotor nuclei. To investigate saccades in detail, oculo-metrics such as latency (response time), velocity, amplitude, frequency response, duration, and error rate are often analyzed (9).

Saccadic eye movement disorders can be categorized, depending on the characteristics of measured oculometrics, to 1) hypokinetic movement disorders often seen in Parkinson’s Disease (PD), Multiple System Atrophy (MSA), Progressive Supranuclear Palsy (PSP), and Corticobasal Degeneration (CBD) and 2) hyperkinetic movement disorders observed in, for example, Huntington’s Disease (HD) and Spinocerebellar Ataxia (SCA) (15). For example, people suffering from PD with mild cognitive impairment (PD-MCI) showed a longer latency in comparison with a control group (8, 14). The saccade latency was associated with the brain regions that were affected in people with PD (9). Those with Alzheimer’s Disease (AD) and amnestic MCI (aMCI) also exhibited significantly longer response time in the anti-saccade task compared to non-aMCI population and healthy controls. More errors were also observed in people with AD and aMCI (11). Obliquely oriented microsaccades were often seen in people with AD and aMCI (8). Most people with MSA presented abnormally large square wave jerks (SWJ) (7, 15). The population with PSP revealed more frequent and larger SWJ, slower saccades, prolonged latencies, and impaired pursuit ocular movement in comparison with the healthy population (7, 13–15). Those with HD also showed dysfunction in fixing their eye stably, impaired initiation and inhibition of saccadic eye movements, and longer response time and decreased velocity in saccades (7, 8, 13, 15). People with SCA manifested unusually large SWJ, slowed saccades, and slightly increased latency of saccades (7, 14). Hence, as described above, saccadic eye movement could be a practical useful biomarker to understand the symptoms of diverse neurological disorders.

There are several methods of eye movement measurement (7, 9, 14). A simple method is to use Frenzel goggles to disable the user from visually fixating on an object while the examiner investigates the eyes of the user. However, since the method does not provide quantitative outcomes, it is not possible to acquire oculo-metrics such as latency of saccadic eye movement. Instead, computer-based recording techniques can quantify ocular movements. Electrooculography (EOG) is one of the traditional techniques and has been employed since the 1970s. Electrodes are placed on the skin around the eyes to record the changes in eye position from the differences in electrical potential between the two electrodes. While EOG provides a good temporal resolution, saccade measurement is sometimes affected by artifacts such as electromyography signals. Another method is to put on an eye directly a modified contact lens with a search coil embedded. When the eye with the search coil moves, a current is induced on the coil and the electrical change can be used to measure the eye position. It is the most accurate method, however, is invasive and painful for the users. Recently, video-based eye tracking is becoming more prevalent. Generally, infrared light is emitted from the light source on the eye tracker to the cornea of eyes. Then, infrared cameras record the positions of reflected light from the cornea and the center of pupil. The relative difference between these two positions is used to calculate the pupil position and gaze direction. The users usually conduct the calibration process to look at several points in the visual field and then the system compares the predetermined points and the measured gaze data to adjust the system configuration depending on the user. The method is non-invasive, offers a high spatial resolution of 0.25° to 0.5°, and provides data of pupillary responses in addition to gaze data. On the other hand, since sampling frequency is a key factor for accurate saccadic eye movement assessment, it will cost more if the eye tracking device with higher sampling frequency is selected. Nonetheless, video-based eye tracking technique is more promising to characterize ocular motions in the absence of absolute biomarkers for ocular assessment.

As the technology of video-based eye tracking has advanced, developers have integrated eye tracking technique into a virtual reality (VR) technology with head-mounted display (HMD) in recent years. The combined system enables us to measure eye movements while showing VR animations. VR is also an effective tool for both diagnosis and intervention in the research field of neurodegenerative disorders (16–18). Previous studies reported that interventions with using VR technology improved motor and cognitive functions of people with disorders such as stroke, MCI, AD, and PD (19, 20). Other studies also found that VR-based measures were more useful as an assessment tool in detecting cognitive impairments and evaluating self-awareness (21–23).

To summarize, the previous studies have found that assessment of saccadic eye movement with video-based eye tracking bears the potential to evaluate various neurodegenerative disorders. In addition, VR technology would have the potential to enhance the diagnosis and interventions of neurodegenerative disorders. We assume that combination of video-based eye tracking technique and HMD-based VR technology could improve the assessment of saccadic eye movement by taking advantages of immersive environments created by VR technology. Therefore, this study aims at investigating a combined device, HTC VIVE Pro Eye, for the purpose of using it for saccadic eye movement assessment and report the measured data of saccadic eye movement and technological findings of the device.

## Materials and Equipment

The main component of this study is the VR-based HMD (HTC VIVE Pro Eye, HTC Corporation). **Table 1** shows the technical specifications of hardware and software components. **Table 1** also lists the main measurement parameters and explains how to interpret the output value of validity of measured eye data. In addition, **Table 2** shows the main technical specifications of the computer used in this study. VR environments are designed on the computer and the VIVE Pro Eye is controlled from the same computer.

**Table 1:** Technical specifications of investigated HTC VIVE Pro Eye.

Item		Specification		
VR headset	Screen	Dual OLED 3.5” diagonal		
	Resolution	1440 x 1600 pixels per eye (2880 x 1600 pixels combined)		
	Refresh rate	90 Hz		
	Field of view	110°		
	Audio	High resolution		
	Input	Dual integrated microphones		
	Interface	USB-C 3.0, DP 1.2, Bluetooth		
	Sensors	SteamVR tracking Accelerometer Gyroscope Proximity Interpupillary distance (IPD) sensor Near-infrared (NIR 850nm) LED (9 for each eye) Infrared camera (1 for each eye)		
Eye tracker	Sampling frequency (binocular)	120 Hz		
	Accuracy (within FOV 20)	0.5°∼ 1.1°		
	Calibration	5 points		
	Trackable field of view	110°		
	Major measurement parameters	Frame sequence Timestamp (ms) Gaze origin (mm) Gaze direction (normalized to between −1 and 1) Pupil position (normalized to between 0 and 1) Pupil diameter (mm) Eye openness (normalized to between 0 and 1) Validity of eye data (The details are below.)		
		Enumerator	Binary digit if valid	Decimal digit if valid
		Gaze origin	00001	1
		Gaze direction	00010	2
		Pupil diameter	00100	4
		Eye openness	01000	8
		Pupil position	10000	16
Software	HTC VIVE Pro Eye setup	version 1.0.8.161		
	HTC software development kit for eye tracking	SRanipal version 1.1.0.1		
	SR Runtime	version 1.1.2.0		
	Steam VR	version 1.11.11		
	Unity, VR design platform	version 2019.2.5f1		

**Table 2:** Technical specifications of computer.

Item	Manufacturer	Model and specification
Computer	Intel	NUC8i7HVK
CPU	Intel	core i7-8809G
Memory	Kingston	ValueRAM SO-DDR4-RAM 2400 MHz 16 GB, SO-DIMM 260 Pins
Storage	Samsung	SSD MZ-V6E500BW, M.2 500GB
Graphic	Intel/AMD	Intel HD Graphics 630/Radeon RX Vega M GH graphics
OS	Microsoft	Windows 10 Education, version 1903

We develop VR environments and implement an eye tracking software algorithm on Unity, following a software development kit (SDK) called SRanipal provided by HTC Corporation. SRanipal includes functions to measure ocular movements and time-related data. The detailed guideline for the program development is found in the SDK; when the SRanipal SDK is installed into a computer, a folder automatically named SRanipal_SDK_1.1.0.1 is created. The guideline is found in the folder: SRanipal_SDK_1.1.0.1\02_Unity\Document\Eye\Document_Unity.html.

**Figure 1:**
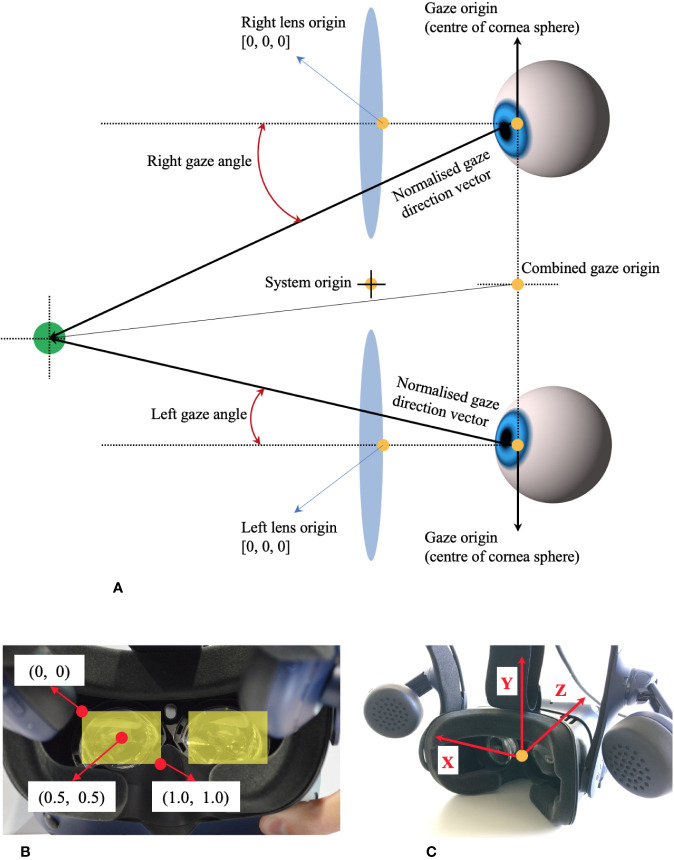
Coordinate system of HTC VIVE Pro Eye, based on the manual of SRanipal SDK. **(A)** Coordinate system of eye tracking on VIVE Pro Eye. **(B)** Coordinate system of pupil position data from user's view. **(C)** Coordinate system of gaze direction vector from user's view.

**Figure 1** shows the coordinate system of VIVE Pro Eye. Recorded data of gaze origin and gaze direction are three-dimensional and based on the right-handed coordinate system. Gaze direction data are normalized to between −1 and 1. Pupil position data are also normalized to between 0 and 1. The origin (0, 0) of pupil position data is at the top left of the sensor area from the user perspective, (0.5, 0.5) is at the center of view field, and (1, 1) is at the bottom right of the sensor area.

## Research Methods

### Study Design

The aim of our study is to evaluate whether the VR headset, HTC VIVE Pro Eye, could be used as an assessment tool for saccadic eye movement. As a first step, we developed a simple VR environment and measurement protocol for saccadic eye movement assessment on Unity design platform, following a previously proposed protocol of saccade evaluation (24), to simulate the environment similar to the design that had been often used on a monitor-based assessment system. Subsequently, we measured saccadic eye movement of healthy young adults. We processed the measured data to calculate oculo-metrics such as peak velocity and latency, analyzed them, and compared the results with those from previous studies. Finally, we summarized the technical findings, limitations, and improvements that were observed in this study. The project was organized at ETH Zurich, Switzerland from August 2019 to February 2020. The ethics was approved by ETH Zurich Ethics Commission (registration number 2019-N-181). We recruited healthy young (between 18 and 35 years old) adults in Switzerland.

### Experimental System

**Figure 2:**
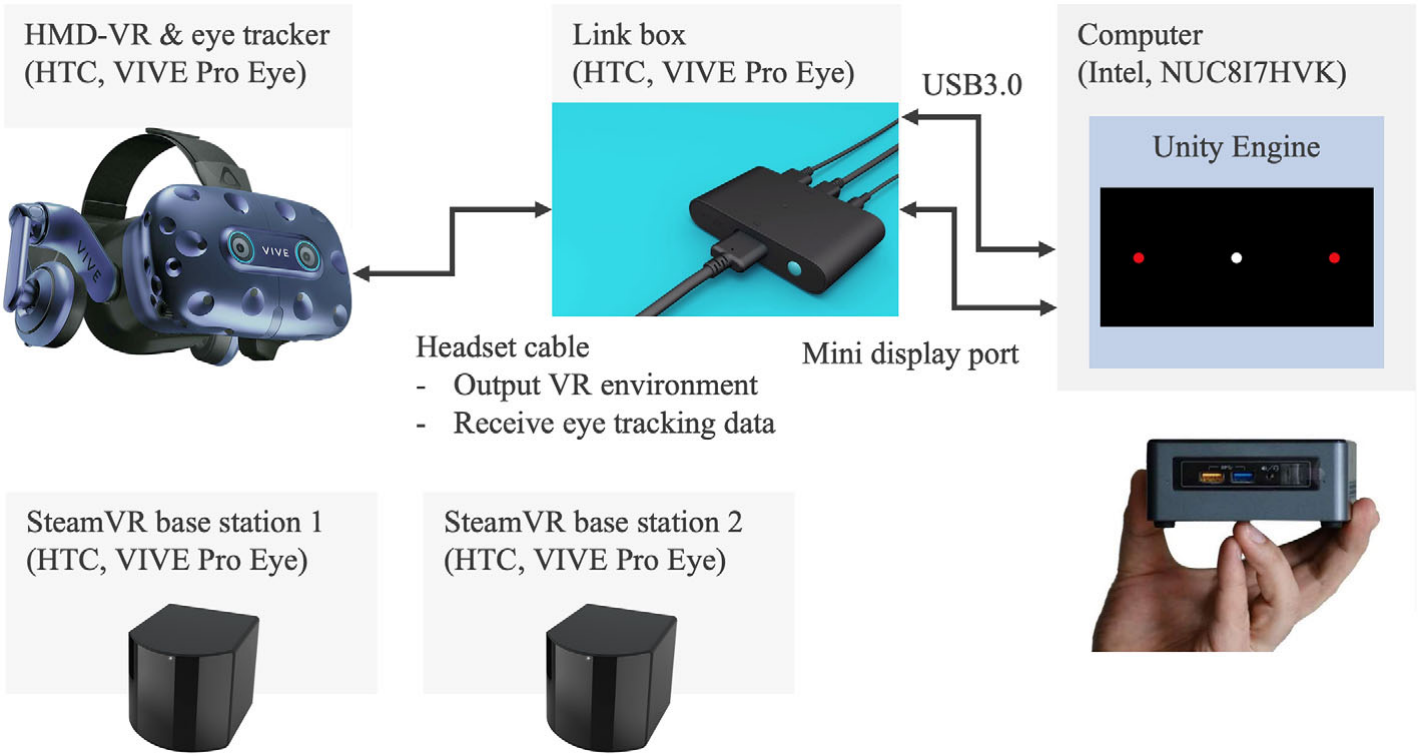
Experiment system of saccadic eye movement assessment with HTC VIVE Pro Eye.

**Figure 2** illustrates the experimental system developed for the assessment of saccadic eye movement using HTC VIVE Pro Eye. The VR environment is designed on the computer of NUC8i7HVK and is output to the VIVE Pro Eye headset *via* the link box. The eye tracker embedded in the VR headset records the ocular movement and the measured data are stored in the computer storage. The base stations are necessary to detect the VR headset.

### Research Protocol

#### Protocol of Saccadic Eye Movement Assessment

**Figure 3:**
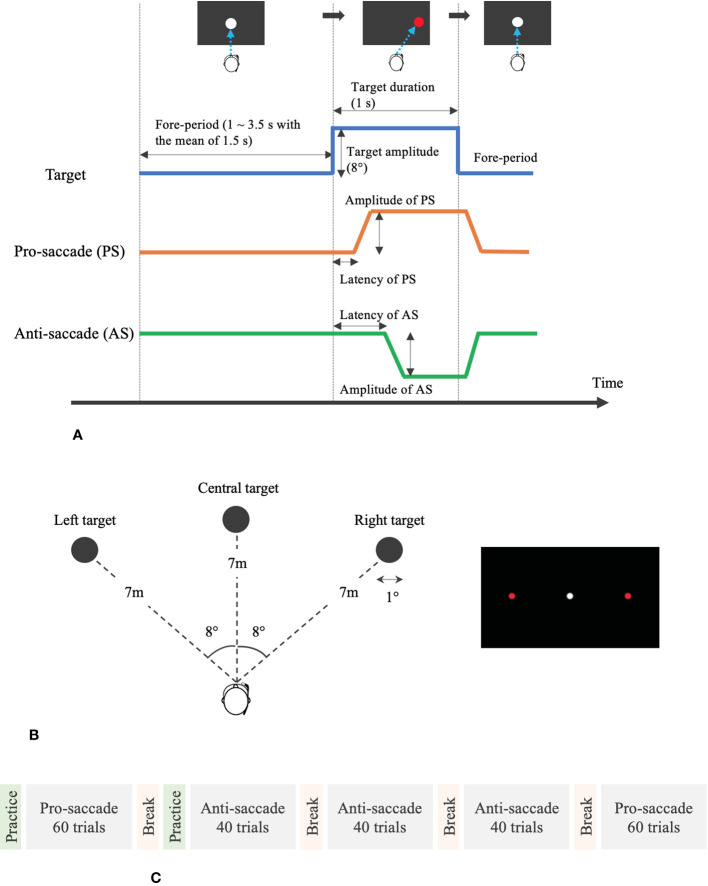
Measurement protocol and VR design for the assessment of saccadic eye movement. **(A)** Protocol of pro- and anti-saccade tasks per trial. **(B)** Designed VR environment for saccade assessment. **(C)** Measurement flow of saccade assessment.

We designed our assessment system of saccadic eye movement, following a previously proposed standardized saccade protocol (24). Oculo-motor scientists and clinicians with diverse experience in saccadic eye movement analysis developed the standardized protocol, with the aim of enhancing the clinical and scientific outcomes. **Figure 3** illustrates the detailed measurement protocol visually, and the following are the points that are recommended in the proposed standardized protocol (24).

Saccade task:
We prepared pro- and anti-saccade tasks in a horizontal direction. Anti-saccade task is a reliable and sensitive measure to evaluate the processes involved in resolving the conflict between volitional and reflexive behavioral responses and can provide important insights on the conditions of various neurodegenerative disorders (25). In general, the subjects look at a target at the center first before performing the saccade tasks. After the target at the center disappears, another target appears on either the right or the left side. The subjects need to move their eyes toward the new target on the side for the case of pro-saccade and toward the opposite direction of the target for the case of anti-saccade.
Protocol of a saccade trial on time domain:
One saccade trial consisted of two phases: 1) a white circle appeared at the center of visual field for between 1 and 3.5 s with the mean of 1.5 s averaged over each set of saccade trials and 2) a red circular target appeared on either the right or the left of the white circle for 1 s.
Time interval between the two phases:
Some measurement protocols set a time interval between after the white target disappears and before the red target appears. However, following the recommendation, we removed the gap phase between these two phases, setting the interval to 0.
Direction and amplitude of stimuli (red target):
As the literature recommends the saccade task in only a horizontal direction and an amplitude of 8°–10° for the red target, we created the VR environment, where the red target came out on the right or left with the amplitude of 8° from the center.
Contrast of targets:
The contrast of the targets was clear enough with over 50%.
Size of targets:
While the proposed protocol recommended the diameter of 0.5° for the system with a screen display, it also stated that the size and shape of the targets were not important. Therefore, we designed the targets with the diameter of 1°. In addition, as shown in **Figure 3**, we set the distance of 7 m between the user’s view and each target. With the predetermined parameters of the distance and the amplitude and size of the targets, we calculated the diameter of targets in meter.
Saccade task flow:
The measurement flow was composed of five main phases. The subjects started with 60 trials of pro-saccade task after practicing the pro-saccade task for 10 times for customization. After a break for 1 min, they practiced anti-saccade task for four times and then moved to 40 trials of anti-saccade task. They performed the second and third anti-saccade tasks consecutively for 40 trials in each with having 1-min short break between the phases and finally ended with the second pro-saccade task for 60 trials. Thus, they conducted 120 trials in each of the pro- and anti-saccade tasks. In each saccade task, the subjects gazed at the red target on the right or left at the equal number of times (i.e., 30 or 20 times on the right and 30 or 20 times on the left for pro-saccade task or anti-saccade task). The fore-period, a duration to display the white target at the center of visual field, varied at random in each saccade trial and the direction of displayed red targets also changed randomly. However, all the subjects experienced the same randomized fore-periods and directions of red targets. The whole measurement took less than 20 min.

#### Programming Algorithm of Eye Tracking

The program was developed using C# programming language on Unity. The programming code is openly published on GitHub: https://github.com/MotorControlLearning, with the detailed explanation of the algorithm for the saccade measurement.

### Data Processing

#### List of Recorded Parameters

We measured the following parameters especially to calculate the important oculo-metrics of latency, peak velocity, and error rate of saccadic eye movement (26). We recorded time information with timestamp in SRanipal SDK and DateTime.Now.Ticks on Unity system. The SDK also provided frame_sequence to record the frame sequence. The following data of ocular movement in each of the left and right eyes were read from the VerboseData in the struct data of ViveSR.anipal.Eye.EyeData_v2 in SRanipal SDK: validity of eye data, eye openness level, pupil diameter, pupil position, gaze origin, and gaze direction. All the data are stored in text files. However, we did not use the data of timestamp because the current version of SRanipal SDK did not provide correct time stamp. The details are described in the following discussion part.

#### Detection Algorithm of Saccades

**Figure 4:**
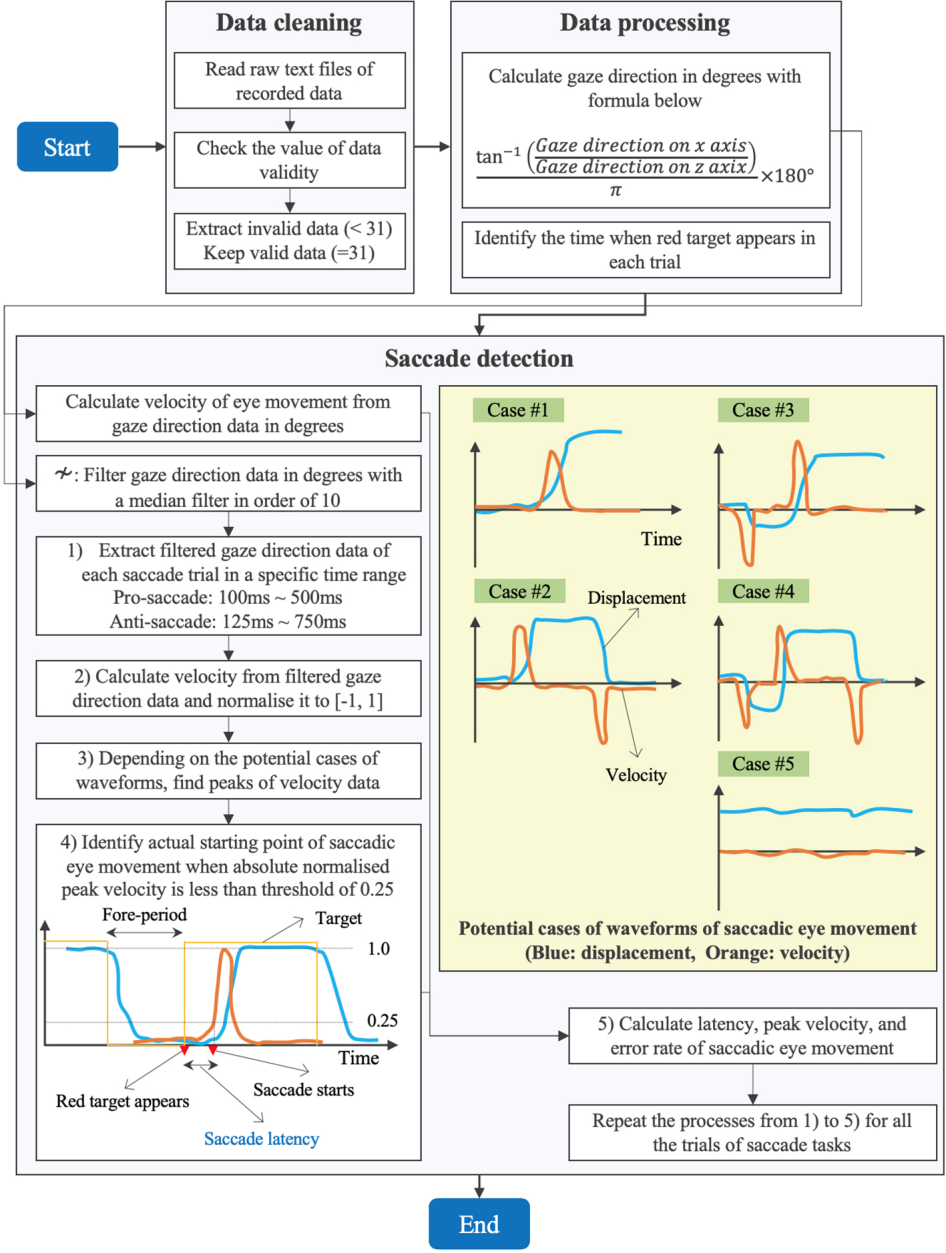
Data processing of eye movement data and detection algorithm of saccades.

**Figure 4** explains the flow of data processing for eye movement data and detection algorithm of saccadic eye movement visually.

First, we performed a data cleaning. After we loaded the raw data of ocular motions from the text files on MATLAB R2019b (MathWorks, MA, U.S.), we checked the value of validity of eye data to eliminate the invalid data with the value less than 31 and kept the valid data with the value equal to 31 (see **Table 1**).

Second, we processed the cleaned data to calculate the gaze direction in degrees with the formula (1). We quantified how far eyes moved from the point of *x* = 0 (see **Figure 1**). Since the recorded data of gaze direction were normalized to between −1 and 1, we calculated the angle in radian by applying the arc tangent and then converted the data from radian to degrees. Here, we have to note that the sign of the calculated data in degrees is negative when eyes move toward the right and is positive when eyes move toward the left (see the coordinate system in **Figure 1**). In addition, we identified the time when the red circular target appeared on either the right or the left in each trial, by reading the Unix time that we recorded in every saccade trial when the red target was displayed.

$$E_{x}=\frac{tan^{-1}\left(\frac{G{\vec{D}}_{x}}{G{\vec{D}}_{z}}\right)}{\pi }180^{\circ }$$

(1) $$E_{x}:\text{Gaze direction in degrees on X axis}$$

$$G{\vec{D}}_{x}:\text{Normalized gaze direction on X axis}$$

$$G{\vec{D}}_{z}:\text{Normalized gaze direction on Z axis}$$

Third, we implemented a program to detect the saccades in each trial. Since spike noise possibly confounded the saccade detection, we removed the spike noise with a median filter with an order of 10 (27). We then extracted the filtered gaze direction data of first saccade trial in a specific time range within the period of 1 s for which the red target was being displayed. Since we knew when the red target appeared based on the Unix time as explained above, we extracted the gaze direction data in a time range from *t_i_*+ 100 to *t_i_*+ 500 ms for the pro-saccade task and from *t_i_*+ 125 to *t_i_*+ 750 ms for the anti-saccade task, where *t_i_*was the time when the *i_th_*(*i* = 1, 2*,…*, 240) red target appeared. We set the time range, assuming that saccadic eye movement would occur in the period by referring to the results of saccade latency in the previous studies (28, 29). Subsequently, we drew velocity from the gaze direction data to understand the time when the velocity changed sharply by inspecting the peaks of velocity as visually explained in **Figure 4**. We used a velocity-based algorithm to detect saccadic eye movements (30). Investigating the waveform of the filtered gaze direction data, we considered five potential cases to detect saccades as illustrated in **Figure 4**. In case #1, gaze at the white central target (fore-period), initiation of saccade, and gaze at the red target are seen. Case #2 observes the return period to move eyes from the red target to the white target in addition to the movement illustrated in case #1. Case #3 is often found in the anti-saccade task because of reflexive eye movement induced by the red target. Thus, another peak velocity could be observed before the correct saccadic eye movement occurs. Similar to case #2, case #4 includes the return phase in addition to the movement in case #3. The final case of #5 is seen if the subjects do not perform the saccadic eye movement task properly. If the gaze direction changed within 1° or 0.001 for normalized pupil position in the inspection period, we applied the case #5. After normalizing the velocity data to between −1 and 1, we found the peaks of velocity that were over the threshold of 0.5 or below −0.5, according to the five cases. We assumed that first velocity peaks in cases #3 and #4 were due to reflexive responses if the signs of peaks did not correspond to those of the predefined protocol. After fixing the peak of saccadic eye movement, we took the absolute value of velocity and set the threshold of 0.25 to define when the saccadic eye movement started in certain response time (i.e., saccade latency) after the red target appeared. Finally, we calculated the latency, peak velocity, and error rate of saccadic eye movement of the trial. We estimated the errors of saccadic eye movement by comparing the sign of peak velocity and the predetermined protocol explaining where the red target appeared in each saccade trial. For example, when the target appears on the right in the pro-saccade task, the peak velocity should be negative, whereas the velocity is supposed to be positive when the target comes out on the left in the visual field. We needed to invert the sign of peak velocity or the target direction of predefined protocol for the analysis of anti-saccade task. We repeated the same procedures explained above for all the saccade trials.

### Data Analysis

Following the algorithm described in the previous sections, we calculated latency, peak velocity, and error rate of saccadic eye movement using gaze direction data. We also evaluated latency and error rate of saccade tasks using pupil position data based on the same saccade detection algorithm explained in **Figure 4**. However, peak velocity was not derived from the pupil position data since the conversion from normalized values to degrees was not available. Specifically, we visualized waveforms of gaze direction and pupil position data on X axis and data distribution of latency, peak velocity, and error rate in box plots. We also compared the calculated data between the data types (i.e., gaze direction data in degrees and normalized pupil position data), between pro- and anti-saccade tasks, and between left and right eyes to evaluate whether the data types, the saccade types, and the individual ocular measurement on each eye affected the results of computed oculo-metrics respectively. The statistical analysis was performed on a data analysis tool of R version 3.6.3. In addition, we investigated the data related to time by visualizing timestamp data recorded with SRanipal SDK and Unix time data recorded on Unity system, in particular to explore the sampling interval of the eye tracking device. Finally, we showed the measured data of pupil diameter as supplementary information.

## Results

### Participants

Seven healthy young adults joined the experiment: four men and three women, 29 ± 4 years old (range 25–36 years).

### Displacement of Gaze Direction in Degrees and Pupil Position

**Figure 5:**
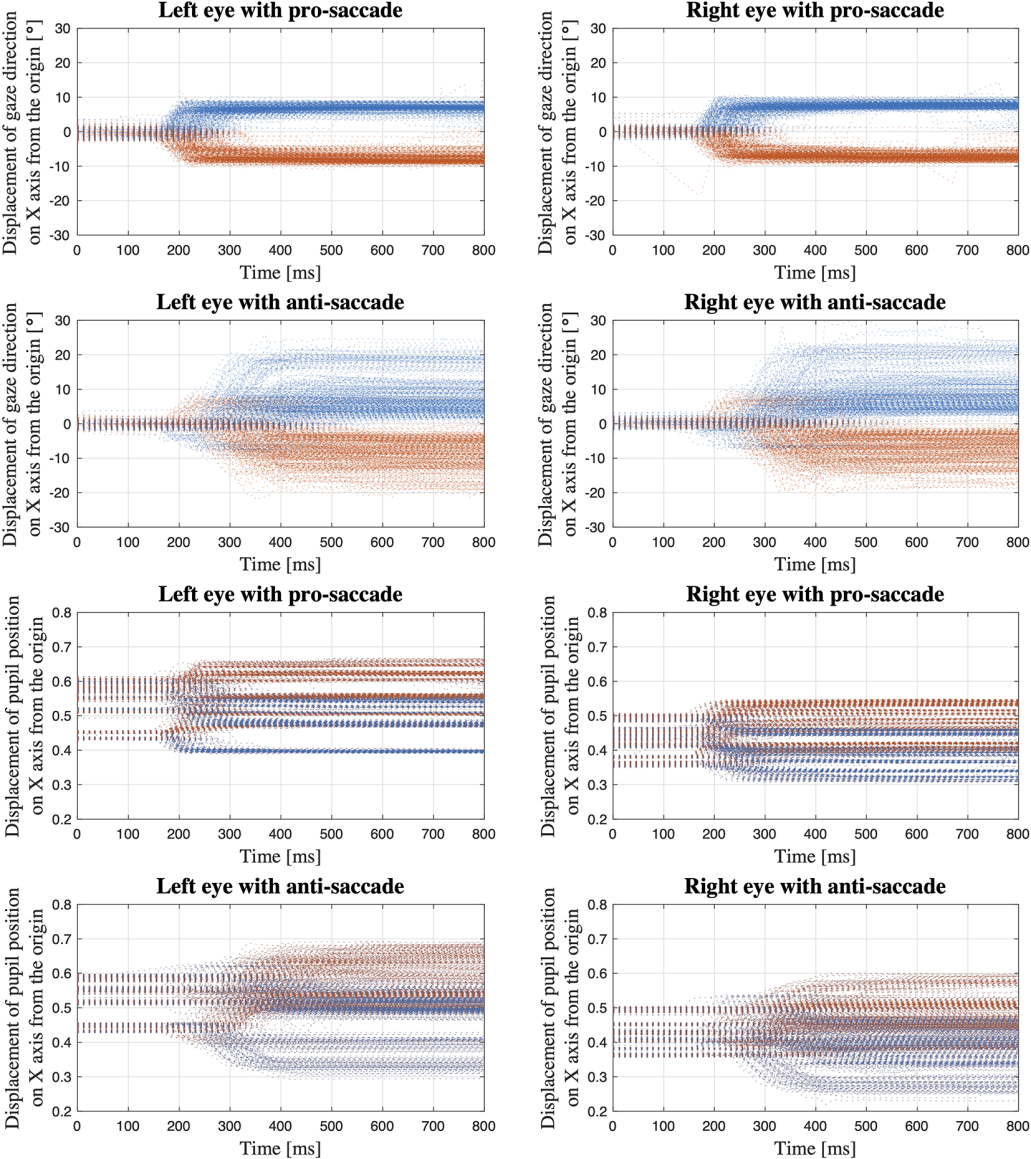
Displacement of gaze direction in degrees and normalized pupil position on X axis from the origin; The data of all the saccade trials of all the participants are overlapped within the same time period from the time when the red target appears to the time 800 ms after the target appears (Blue dots: gaze toward the left; Orange dots: gaze toward the right).

**Figure 5** shows the displacement of gaze direction on X axis from the origin (see **Figure 1** for the coordinate system of VIVE Pro Eye). Separating the processed data depending on eyes (i.e., left and right), saccade tasks (i.e., pro- and anti-), and data types (i.e., gaze direction in degrees and normalized pupil position), we extracted each saccade trial of each participant and overlap each displacement waveform of each trial in a single figure.

### Oculo-Metrics of Latency, Peak Velocity, and Error Rate of Saccadic Eye Movement, and Pupillary Response

**Figure 6:**
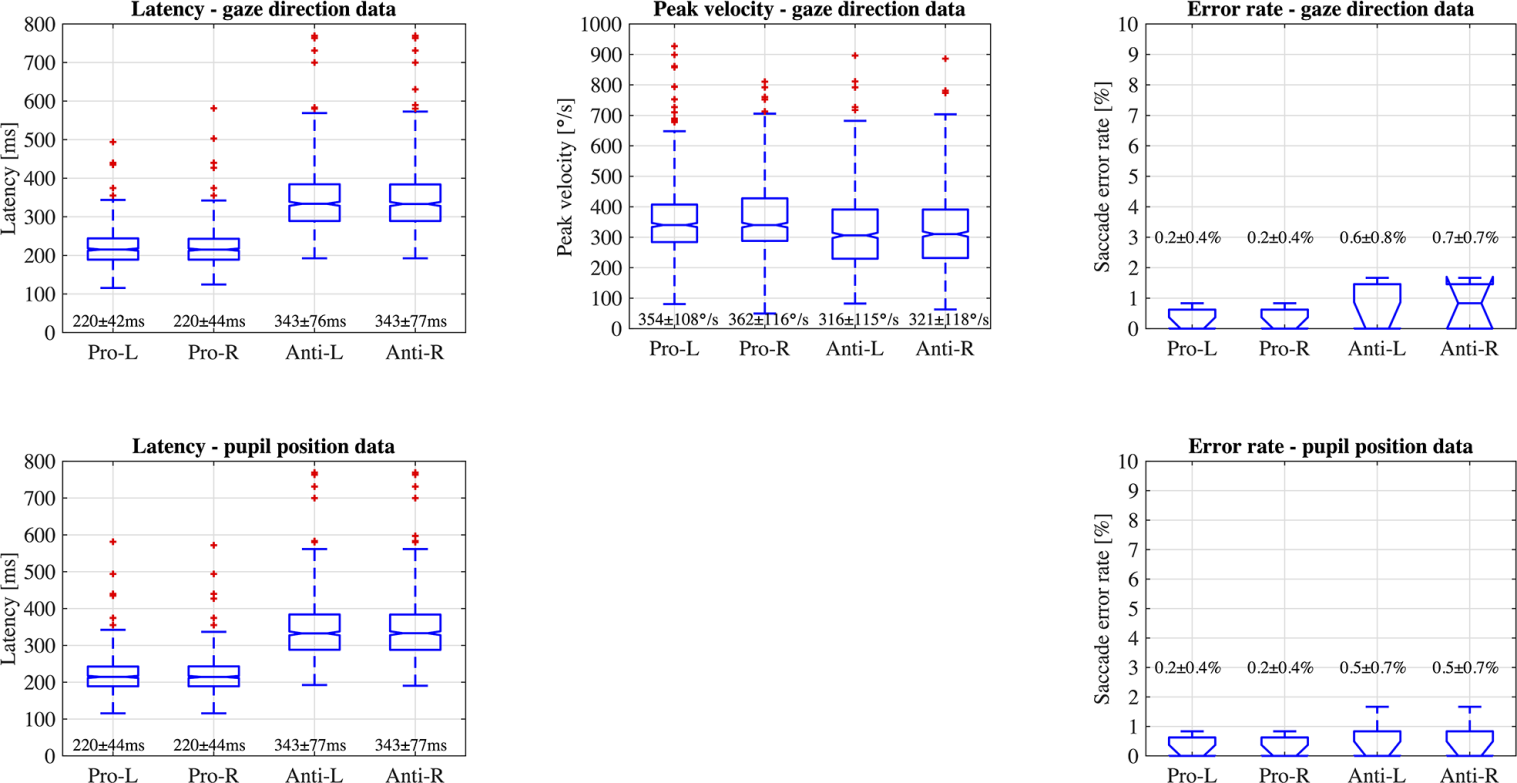
Data distribution of parameters; latency, peak velocity, and error rate of saccadic eye movement of each eye in each pro- and anti-saccade task of all the participants (Pro, Pro-saccade; Anti, Anti-saccade; L, Left eye; R, Right eye).

**Figure 7:**
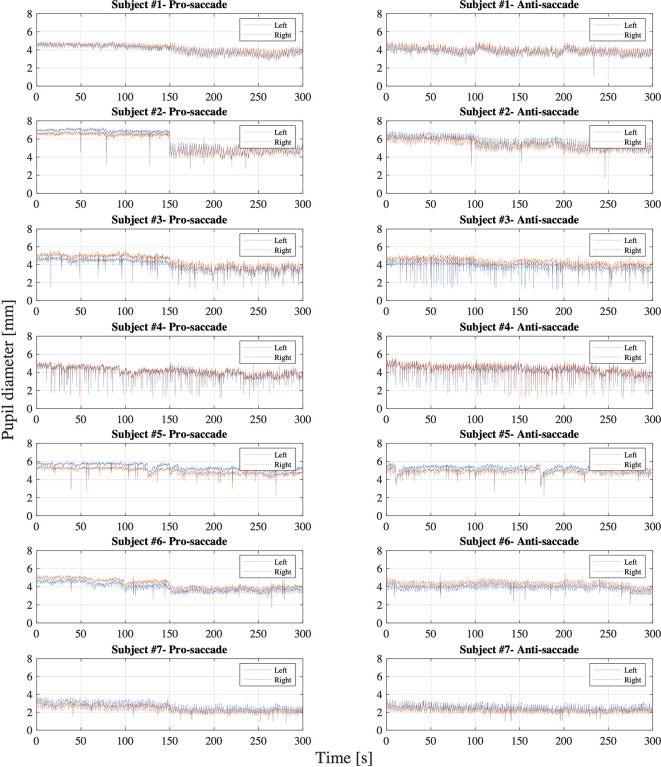
Pupil diameter of each left and right eye in each pro- and anti-saccade task of all the participants.

**Figure 6** illustrates the data distribution of the parameters: latency, peak velocity, and error rate of each pro- and anti-saccade task of each eye from all the participants. The first row of the figure is the result from gaze direction data in degrees and the second row is the result from normalized pupil position data. **Figure 7** shows the changes of pupil diameter of left and right eyes during each pro- and anti-saccade task in each participant. Each sub-figure illustrates the changes of pupillary response over the 120 saccade trials. **Table 3** shows the mean and standard deviation (SD) of each ocular parameter averaged over all the participants.

**Table 3:** Oculo-metrics of saccadic eye movement and pupillary response.

Parameters		Mean ± SD	
		Pro-saccade	Anti-saccade
Gaze direction data: Left eye	Latency	220.36 ± 43.31 [ms]	343.29 ± 76.22 [ms]
	Peak velocity	353.64 ± 108.02 [°/s]	316.15 ± 115.49 [°/s]
	Error rate	0.24 ± 0.41 [%]	0.60 ± 0.79 [%]
Gaze direction data: Right eye	Latency	220.45 ± 44.01 [ms]	343.41 ± 76.67 [ms]
	Peak velocity	362.15 ± 115.73 [°/s]	321.43 ± 117.89 [°/s]
	Error rate	0.24 ± 0.41 [%]	0.71 ± 0.75 [%]
Gaze direction data: Both eyes combined	Latency	220.40 ± 43.16 [ms]	343.35 ± 76.42 [ms]
	Peak velocity	357.90 ± 111.99 [°/s]	318.79 ± 116.69 [°/s]
	Error rate	0.24 ± 0.41 [%]	0.66 ± 0.76 [%]
Pupil position data: Left eye	Latency	220.08 ± 43.82 [ms]	342.55 ± 76.67 [ms]
	Error rate	0.24 ± 0.41 [%]	0.48 ± 0.66 [%]
Pupil position data: Right eye	Latency	220.05 ± 43.75 [ms]	342.60 ± 76.73 [ms]
	Error rate	0.24 ± 0.41 [%]	0.48 ± 0.66 [%]
Pupil position data: Both eyes combined	Latency	220.07 ± 43.77 [ms]	342.58 ± 76.67 [ms]
	Error rate	0.24 ± 0.41 [%]	0.48 ± 0.66 [%]
Pupillary data: Left eye	Pupil diameter	4.30 ± 1.15 [mm]	4.21 ± 1.04 [mm]
Pupillary data: Right eye	Pupil diameter	4.29 ± 1.08 [mm]	4.22 ± 0.97 [mm]

### Sampling Interval of Eye Tracking

**Figure 8:**
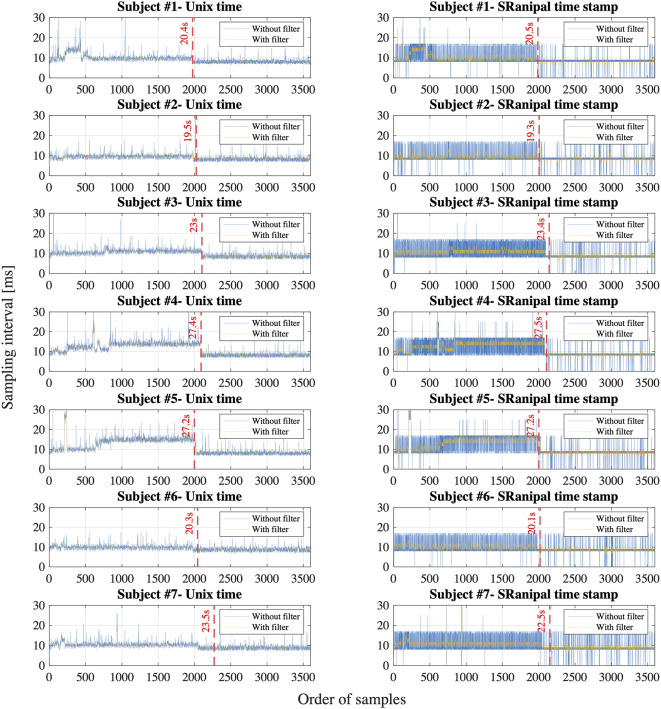
Sampling interval calculated with Unix time on Unity and time stamp from SRanipal SDK of all the participants; Blue line shows the unfiltered data and yellow line shows the filtered data.

**Figure 8** shows the sampling interval of eye tracking recorded with Unix time on Unity and with time stamp from SRanipal SDK of each participant. We particularly visualized the sampling interval of the first 3600 samples that we recorded; we assumed 30 s of recording time at the sampling frequency of 120 Hz. X axis is the order of samples and Y axis is the sampling interval in milliseconds between the samples. Each sub-figure shows two types of data: raw data and filtered data. We filtered the data of Unix time with a median filter with an order of 10 and the data of SRanipal time stamp with a moving average filter with a window size of 5. We then estimated the point where the sampling interval became smaller than 8 ms, as highlighted with red dash lines, by using the filtered data.

## Discussion

### Displacement Data of Gaze Direction and Pupil Position

The first four sub-figures in **Figure 5** visualize the displacement of gaze direction in degrees both for each left and right eye in each pro- and anti-saccade task of all the participants. A clear difference between the eyes is not observed in both saccade tasks. On the other hand, we see differences between the saccade tasks visually. While the subjects moved their eyes almost precisely toward the red target at ±8° from the origin that was positioned as designed in **Figure 3** in the pro-saccade task: 7.3 ± 1.3° for left eye, 7.4 ± 1.2° for right eye, we observe a larger variability ranging from around ±3° to ±20° in the anti-saccade task: 8.3 ± 4.8° for left eye, 8.2 ± 4.9° for right eye. In addition, we visually see longer latency and more reflexive responses between 200 and 300 ms in the anti-saccade task compared to pro-saccade task; latency is 220.40 ± 43.16 ms in the pro-saccade task and 343.35 ± 76.42 ms in the anti-saccade task, and reflexive responses are observed for 1.4% of all the trials from all the participants in the pro-saccade task and for 13.2% in the anti-saccade task when we define the reflexive response as a movement of eyes that shift from the central white target toward the erroneous direction over 3°. The reflexive response is usually observed in anti-saccade task because we automatically respond to the red stimulus target when it appears, despite being aware that we need to gaze at the opposite direction to the target (31).

The last four sub-figures show the displacement of normalized pupil position. When the waveforms are compared between the eyes, the values are generally larger in the left eye: the data range from 0.4 to 0.65 in the pro-saccade task and from 0.3 to 0.68 in the anti-saccade task for the left eye, whereas we observe the data between 0.33 and 0.58 in the pro-saccade task and between 0.22 and 0.6 in the anti-saccade task for the right eye. Moreover, when we look at the waveforms in the first 100 ms, we see the pupil position varies depending on the participants in comparison with the gaze direction data. These may be caused by a computational process embedded in the SRanipal SDK to convert the raw gaze data to the normalized pupil position data. Similar to the gaze direction waveforms, the pupil position waveforms also illustrate larger variability and longer latency in the anti-saccade task, although the reflexive responses are less distinguishable.

### Latency, Peak Velocity, and Error Rate of Saccadic Eye Movement

#### Comparison Between Data Types, Saccade Types, and Eyes

We statistically compared the calculated data of latency and error rate of saccadic eye movement between two data types: gaze direction data type in degrees and normalized pupil position data type. Wilcoxon signed-rank test showed no significant difference at the significance level of 5% between the data types: *P* = 0.75 for latency, *P* = 0.98 for SD of latency, and *P >* 0.999 for error rate in the pro-saccade task, and *P* = 0.73 for latency, *P* = 0.77 for SD of latency, and *P* = 0.56 for error rate in the antisaccade task. In addition, we found significant differences between pro- and anti-saccade tasks in latency (*P <* 0.001), SD of latency (*P <* 0.001), and peak velocity (*P <* 0.001), but not in SD of peak velocity (*P* = 0.77) and error rate (*P* = 0.07). We also compared the data between left and right eyes, finding no significant differences: *P* = 0.97 for latency, *P* = 0.99 for SD of latency, *P* = 0.16 for peak velocity, *P* = 0.51 for SD of peak velocity, and *P* = 0.82 in error rate. Thus, if latency and error rate are main oculo-metrics in saccadic eye movement assessment, both of gaze direction data in degrees and normalized pupil position data can be used. Only gaze direction data can compute peak velocity of saccades.

#### Comparison With the Results of Previous Studies

Previous studies investigated saccadic eye movement of healthy populations at different ages using a 2D monitor-based assessment system. We compared our results with those studies.

Pro-saccadic eye movement was assessed in 100 healthy subjects at the age between 6 and 76 years, using an infrared video-based eye tracking technique with a sampling frequency of 220 Hz (28). The stimulus target was shown at ±5°, ± 15°, and ±30° horizontally. The criteria of saccade detection were 1) the velocity was over ±100°*/*s, 2) the evaluation duration was 500 ms after the stimulus target appeared, and 3) the amplitude was more than 0.5 of the relevant target displacement. The result showed that the latency of pro-saccade task ranged approximately from 110 and 260 ms for the population at the age between 20 and 39 years. Peak velocity increased as the position of target was placed farther from the center of visual field. The peak velocity was almost between 80 and 290°*/*s when the target appeared at ±5°, whereas the velocity ranged from 250 to 500°*/*s when the target was positioned at ±15°. Another study also researched age-related changes of eye movement in 250 healthy people at the age between 18 and 70 years, using an infrared-based video-oculography (VOG) (32). The study defined the criteria of saccade detection: 1) the amplitude of eye movement was over 10°, 2) the evaluation duration was within 500 ms after the appearance of stimulus target, and 3) the saccade amplitude was over 10% of the target amplitude. The study found that the latency of pro-saccade task was 237.24 ± 18.23 ms in the leftward saccade and 265.34 ± 35.84 ms in the rightward saccade for the subjects at the age between 18 and 30 years (Group A), and was 241.44 ± 24.80 ms in the leftward saccade and 252.26 ± 37.65 ms in the rightward saccade for the population group at the age between 31 and 40 years (Group B). The research also reported the peak velocity: 245.30 ± 78.20°*/*s in the leftward saccade and 237.52 ± 75.64°*/*s in the rightward saccade for the Group A and 228.28 ± 66.29°*/*s in the leftward saccade and 226.50 ± 67.06°*/*s in the rightward saccade for the Group B.

On the other hand, another study investigated both pro- and anti-saccadic eye movement tasks in 1,058 healthy young adults at the age between 16 and 40 years, with an infrared-based oculography recording the ocular motions at 1 kHz (33). The saccade task started with presenting a target at the center of visual field for a random period between 500 and 1,500 ms and then a stimulus target at one of the ten horizontal positions: ± 3°, ± 6°, ± 9°, ± 12°, and ±15° for 600 ms in the pro-saccade task and 1,000 ms in the anti-saccade task. The pro-saccade task consisted of 200 trials in total and the anti-saccade task consisted of 50 trials. After the recorded data were filtered with a 300 Hz low-pass filter, the saccade in each trial was detected based on both eye acceleration and eye velocity criteria. Specifically, the presence of saccade was detected if the eye acceleration data exceeded a threshold: six times the median value of the SD of the acceleration data in the first 80 ms of all the trials in each person, or if the absolute value of eye velocity exceeded 50°*/*s. Then, the study defined borders of the saccades as the areas where the eye velocity was less than three times the median value of the SD of the eye velocity data measured in the first 80 ms of all the trials in each participant. The research observed the latency of 177.2 ± 18.52 ms (range: 142 ∼ 322 ms) for the pro-saccade case and of 305.5 ± 43.06 ms (range: 113 ∼ 539 ms) for the anti-saccade case. The error rate of the anti-saccade task was 37.7 ± 21.5%. Saccadic eye movement was also inspected with an EOG device recording ocular movement at 500 Hz sampling frequency in 168 healthy subjects at the age between 5 and 79 years (34). The study arranged two different types of saccade task conditions: 1) overlap condition where a target appearing at the center of visual field remained illuminated when a stimulus target appeared and 2) gap condition where after the central target disappeared, no target was shown for 200 ms and then the stimulus target came out. In each condition, the stimulus target appeared at ±20° from the center and remained illuminated for 1 s. 120 trials of pro-saccade test and 240 trials of anti-saccade test were tested. The saccade was detected when the eye velocity exceeded 30°*/*s in the evaluation duration between 90 and 1,000 ms. The results revealed that the mean latency of saccadic eye movement over the participants was 224.71 ms for the pro-saccade and 307.14 ms for the anti-saccade in the gap condition, while the mean latency was 280.01 ms for the pro-saccade and 357.77 ms for the anti-saccade in the overlap condition. However, the latency was clearly shorter for the young population in the study. The error rate of anti-saccade task ranged from 0 to 48% for the participants at the age between 20 and 40 years. Moreover, a regression analysis was performed in 327 healthy subjects aged between 9 and 88 years, using an infrared reflection devise (35). The study measured both pro- and anti-saccadic eye movement for 200 trials in each. Overlap condition was utilized in the pro-saccade test where 1.2 s after a central fixed point was shown in the middle of a monitor, a stimulus target appeared at ±4° from the center and then remained brightened for 1 s. Gap condition was used in the anti-saccade test where the central target disappeared 0.2 s before the onset of the stimulus target and then the stimulus target remained visible for 1 s. Saccade onset was defined by an eye velocity threshold of 20°*/*s and latency of saccadic eye movement was computed in the time range of 136 ∼ 700 ms. In addition, pro-saccadic eye movement during the anti-saccade task was considered as an error if the latency of eye movement was longer than 80 ms. The study results indicated that the latency ranged nearly from 125 to 290 ms (SD: 18 ∼ 102 ms) for the pro-saccade task and from 150 to 380 ms for the anti-saccade task in the participants aged between 25 and 37 years.

In summary, while the prior studies discussed above used different measurement protocols in terms of position of stimulus target, configuration on time domain (i.e., gap or overlap condition, duration of illumination of targets on monitor), saccade detection algorithm, sampling frequency of eye tracking device, monitor specification, target population, and sample size, the descriptive statistics show that our results of latency, peak velocity, and error rate are within the range of or close to the results of these previous studies. Therefore, this seems to indicate that the HTC VIVE Pro Eye could be useful as an assessment tool of saccadic eye movement for the specific oculo-metrics.

### Pupillary Response

On the whole, **Figure 7** shows that the pupil diameter did not fluctuate widely during each pro- and anti-saccade task for both eyes, while we observe some differences between the participants. Specifically, subject #7 showed smaller pupil diameter than the others. This may be because subject #7 wore glasses during the measurement. Iris color might not cause the differences in pupil diameter between the participants as reported in (36). A previous research measured changes of pupil diameter under the different luminance conditions in 155 healthy people with the mean age of 29.7 ± 17.8 years and the age range of 6 ∼ 64 years (37). The measurement was conducted under four different illumination levels: scotopic (0.1 cd*/*m^2^), mesopic (1 cd*/*m^2^), low photopic (10 cd*/*m^2^), and high photopic (100 cd*/*m^2^) visions. The study observed the pupil diameter of 5.4 ± 0.7 mm and 3.9 ± 0.4 mm for the subjects aged between 21 and 30 years and of 4.4 ± 0.5 mm and 3.5 ± 0.5 mm for the subjects aged between 31 and 40 years in mesopic and low photopic visions respectively. Another study also inspected the changes of pupillary response at five luminance levels: 0, 0.5, 4, 32, and 250 cd*/*m^2^ in 245 healthy population (mean age: 51.9 ± 18.3 years; age range: 6 ∼ 87 years) (38). The research found a mean pupil diameter of 5.39 ± 1.04 mm at 0 cd*/*m^2^, 5.20 ± 1.00 mm at 0.5 cd*/*m^2^, 4.70 ± 0.97 mm at 4 cd*/*m^2^, 3.74 ± 0.78 mm at 32 cd*/*m^2^, and 2.84 ± 0.50 mm at 250 cd*/*m^2^ and decreasing pupil size with increasing age. Moreover, a different research evaluated reliability of pupil diameter measurement in 416 healthy participants across diverse demographics with the mean age of 42 ± 8.7 years and the age range from 18 to 73 years (39). The research used an infrared eye tracking technique with a 2D monitor with 85 cd*/*m^2^ luminance in a room at 344 cd*/*m^2^ luminance level, revealing that the mean pupil diameter was 3.53 ± 0.26 mm for right eye and 3.54 ± 0.28 mm for left eye and that the test-to-test reliability was strong. When we compare our experiment results (see **Table 3**) with those in the previous studies mentioned above, we conclude that the eye tracking device in HTC VIVE Pro Eye also measures pupillary response properly. While it is difficult to compare the results precisely, our data are in the range of or close to the measured mean pupil diameter of the prior studies. Future studies should, however, determine the test-retest reliability of our approach in predefined target populations to substantiate this assumption.

### Sampling of Eye Tracking

The eye tracker on VIVE Pro Eye samples eye movement at the maximum frequency of 120 Hz. When ocular motions are sampled at 120 Hz, the sampling interval is 8.33 ms. We report the following findings related to the sampling of the eye tracker.

**Figure 9:**
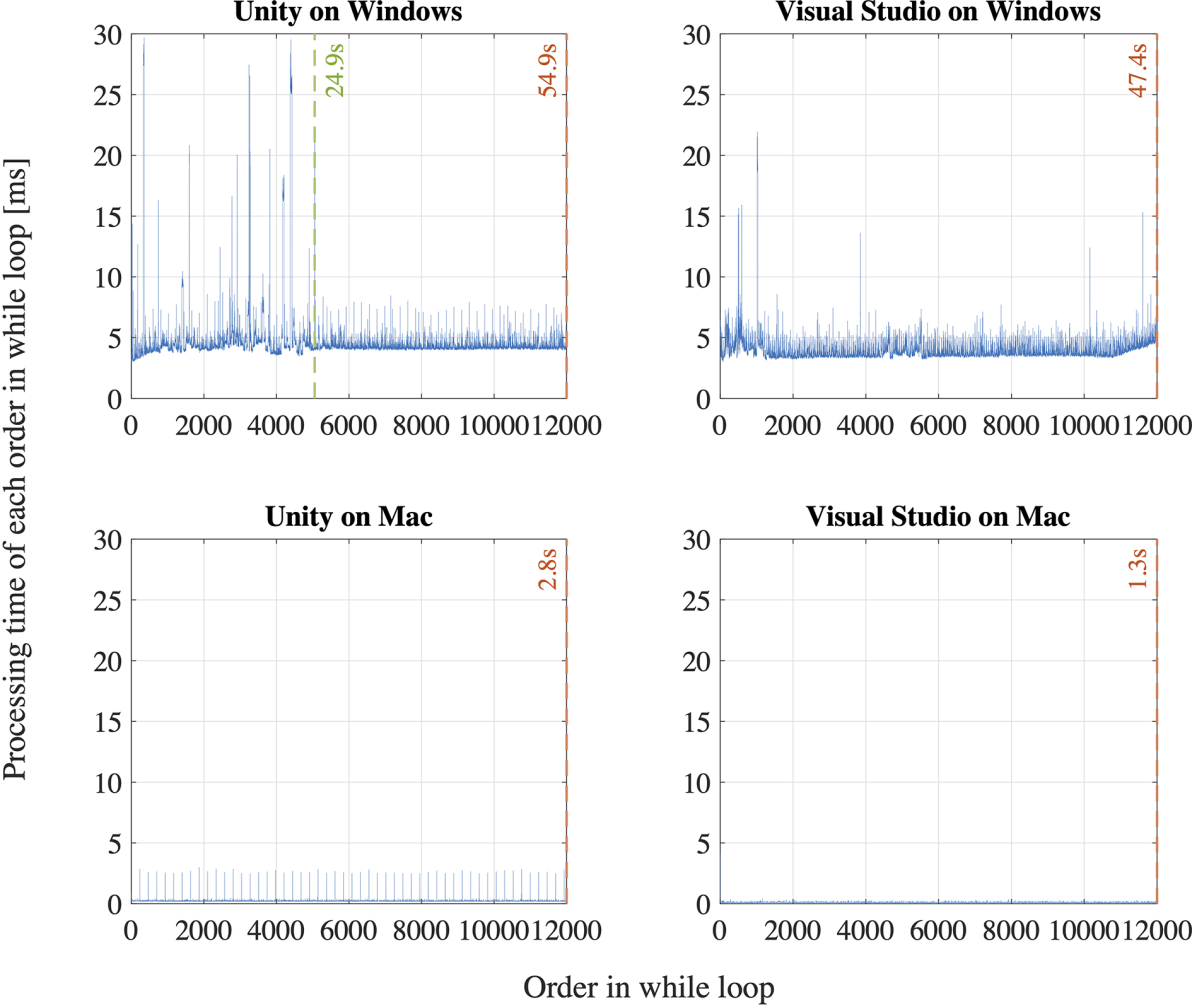
Evaluation of a while loop for 12,000 times on Unity and Visual Studio on Windows and Mac operating systems.

First, we observe that the sampling interval is longer than 8.33 ms for most of the first 2,000 ∼ 3,000 samples for all the participants in both time measurement parameters: Unix time and SRanipal time stamp as shown in **Figure 8**. Specifically, we find that the sampling interval decreased to the expected value of around 8 ∼ 9 ms, 19 ∼ 28 s after the sampling started as highlighted with the red dash lines in **Figure 8**. The time when the sampling interval changes varies depending on the participants, though the timing is similar or quite close between the two time measurement parameters. We suppose that the longer sampling interval may occur because of processing speed of C# programming on Unity. As explained on our GitHub website: https://github.com/MotorControlLearning we recorded eye movement with using the callback function. More specifically, we implemented a while loop in the callback function to keep recording ocular motions while the saccadic eye movement task was performed. Assuming that the processing speed of the while loop might cause the longer sampling interval (40), we further investigated the computation of while loop. We created a simple while loop on C# programming and measured the processing speed of the simple while loop of 12,000 times in four different configurations: 1) Unity on Windows operating system (OS) that was used in the measurement of saccadic eye movement in this study, 2) Visual Studio on Windows OS (without Unity), 3) Unity on Mac OS (Apple MacBook Pro, Retina, 15-inch, Late 2013, 16-GB memory, 2.3-GHz Quad-Core Intel Core i7-4850HQ), and 4) Visual Studio on Mac OS. As a result, we found that the processing time of the simple while loop was longer than 8.33 ms for the configuration of Unity on Windows OS at some samples in the first 29.4 s after the program was executed (see **Figure 9**). In addition, we observe that the processing speed decreased to less than 8 ms, 29.4 s after the while loop program started. On the other hand, while the longer processing time than 8.33 ms was also observed for the configuration of Visual Studio on Windows OS, we saw the situation less frequently than the configuration of Unity on Windows OS as the total processing time explained: 54.9 s for Unity on Windows OS and 47.4 s for Visual Studio on Windows OS. Interestingly, we discovered that the processing time was much shorter on Mac OS than Windows OS as shown in **Figure 9**. The processing time of each while loop was less than 5 ms for the configurations with Mac OS and the total processing speed was 2.8 s for Unity on Mac OS and 1.3 s for Visual Studio on Mac OS despite that the technical specification of the Mac laptop was lower than the computer with Windows OS. Since we needed to use the computer with Windows OS to provide enough power to support HTC VIVE Pro Eye and to sample eye movements at the maximum sampling frequency of 120 Hz, we intentionally had a non-assessment period over 30 s after the recording of eye movement started to wait for the sampling interval to get close to the expected value of 8.33 ms. Therefore, we recommend setting the non-assessment period for over 30 s in the initial measurement phase if Windows OS is used and the maximum sampling of 120 Hz is necessary. However, it is also important to note that the duration of 30 s may not be enough occasionally since we have observed that the longer processing time continued even after the 30 s with around 3 ∼ 5% possibility. If a laptop with Mac OS meets the requirements of HTC VIVE Pro Eye, the issue would not be encountered, though we recommend evaluating whether the problem occurs on any configurations.

**Figure 10:**
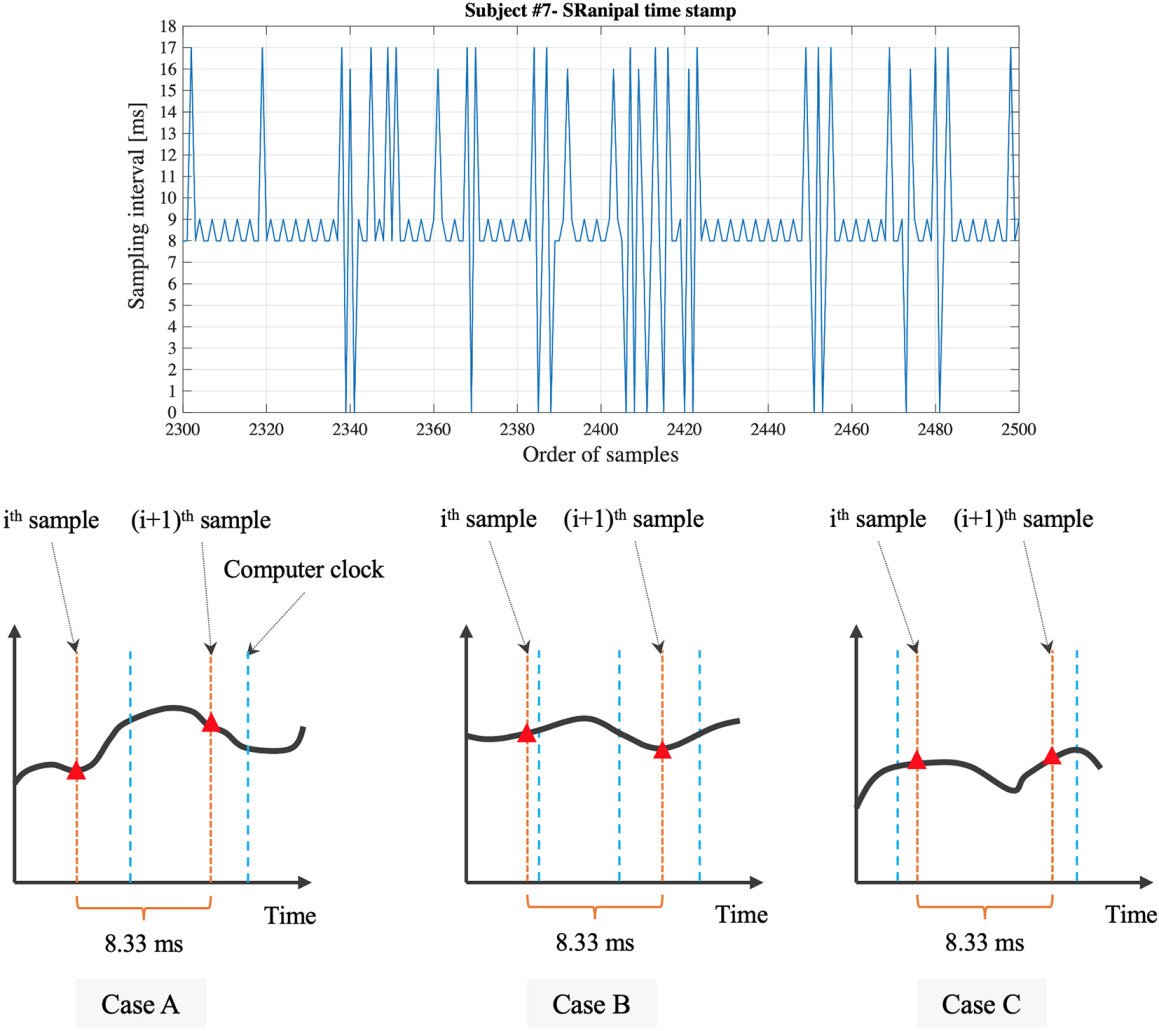
Detailed sampling interval based on the time stamp recorded with SRanipal SDK and visual explanation of relation between sampling of eye tracker and clock signal of computer.

Second, we find that the time stamp recorded in SRanipal SDK sometimes becomes zero while the time recorded with Unix time does not reach zero as visually confirmed in **Figure 8**. We consider two reasons for the finding. First reason is a software bug of SRanipal SDK. When we inspected the recorded data of time stamp, frame, and pupil diameter specific to two consecutive samples, we found that the pupil diameter of left eye changed from 4.857666 to 4.847290 mm and the pupil diameter of right eye also changed from 4.549805 to 4.549103 mm, whereas the values of time stamp and frame did not change. If the time stamp value had been the same for these two samples, the same pupillary response should have been recorded. We reported the finding on HTC developer community forum and HTC confirmed the problem as of the 3rd of December in 2019. Due to the issue, we did not use the time data of time stamp recorded by SRanipal SDK in the data analysis of saccadic eye movement and used the data of Unix time instead. HTC plans to fix the issue in the next version of SRanipal SDK. Second reason is processing speed of C# programming on Unity. As illustrated in **Figure 9**, the processing time measured with the configuration of Unity on Windows OS fluctuates between 3.9 and 8.5 ms. Since the sampling interval of eye tracker is 8.33 ms, it is possible for the computer to record the same sample twice in the while loop (40). For instance, if the computer records the data of eye movements in the processing time of 4 ms, the computer possibly activates the clock signal twice within the period of the sampling interval of eye tracking, 8.33 ms, as illustrated in Case B of **Figure 10**. This means that the computer records the same sample (*i_th_*sample in Case B of **Figure 10**) twice continuously. The situation happens more frequently especially when the timing of clock activation and the sampling of eye tracker are close to each other. To summarize, if the next version of SRanipal SDK solves the issue of time stamp and we eliminate the duplicated data due to recording the same sample twice, we could utilize the time stamp data recorded by SRanipal SDK for the data analysis of saccadic eye movement with more accuracy. A consequence of our observation is also related to the replicability of existing research. It seems important for researchers to pay attention to the hardware configuration of their experimental set-up when one of the aims of their research is to replicate findings that were previously reported for saccadic eye movement behaviors.

Third, while the time stamp recorded by SRanipal SDK needs to be improved as mentioned above, we notice that the time stamp values fluctuate; the calculated sampling interval from the time stamp data recorded with SRanipal SDK becomes 8, 9, 16, or 17 ms as a portion of the data of subject #7 shows in **Figure 10**. We subcategories these four values into two sets: 1) 8 and 9 ms, and 2) 16 and 17 ms. We suppose that the two sets are observed because of the relation of timing between the sampling of the eye tracker and the clock signal of the computer (40). In particular, it is possible to see the first set of 8 and 9 ms if Case A occurs as explained in **Figure 10**, while we possibly encounter Case C where we cannot record the *i_th_*sample because of longer processing time of the computer than the sampling interval of 8.33 ms, causing the calculated sampling interval to become 16 or 17 ms. In addition, we see the difference of 1 ms in each set. The difference might be caused by the computational calculation of time stamp data of Tobii eye tracker as discussed in the white paper published from Tobii Technology (41). Although the paper refers to previous Tobii products using an eye tracker with sampling frequency of 60 Hz, it states that there is the uncertainty in the time stamp data with a nominal value of 1 ms. Therefore, if the Tobii eye tracker integrated in HTC VIVE Pro Eye also has the similar uncertainty, we could also observe the 1 ms difference in our measured data.

In this section, we have reported our findings regarding the sampling technique of eye tracking used in HTC VIVE Pro Eye. From what we have investigated, we recommend evaluating the capability of sampling of VIVE Pro Eye in the development phase especially if temporal parameters of eye movement are investigated and the maximum sampling frequency at 120 Hz is required. Specifically, if the similar experimental system to ours using Unity on Windows OS is used, we would suggest setting a non-assessment period more than 30 s after the eye tracker starts to record. The waiting could lead the system to record the data at the interval of 8.33 ms more frequently. Nonetheless, the non-assessment period may not be needed depending on the OS and hardware configuration of the computer. Finally, since the next version of SRanipal SDK is going to fix the problem of time stamp data as explained above, we propose trying the next version when it is released. If the new version provides the time stamp data correctly, we could improve the precision of the calculated oculo-metrics of saccadic eye movement by using the time stamp instead of Unix time because the time stamp data are supposed to provide more accurate time data with considering the image delivery time from the tracking sensor to the eye tracker firmware as discussed in Tobbi Technology (41).

### Limitations and Improvements

#### Measurement Protocol of Saccadic Eye Movement Assessment

The main purpose of this study was to evaluate whether HTC VIVE Pro Eye could be used as an assessment tool of saccadic eye movement. While we have observed that the device can function as an assessment tool by comparing our results with the descriptive statistics of previous studies, one of the aspects in our study that could be perceived as a limitation is that our sample size is small and the age-group is specific to a young and healthy population. The assessment ultimately targets individuals prone to develop cognitive impairments that expectedly are older than the assessed convenience sample. In these elderly people, issues of attitudes and acceptance with VR headsets might result in differing findings. In that sense, our results are first indicative findings that should be replicated with older individuals. Recent research revealed, however, that the approach of wearing VR headsets seems feasible also in older adults with cognitive and/or physical impairments (42). Further measurement with an increasing sample size and diverse demographic groups, and the direct comparison with the data obtained with a highly reliable and high-spec VOG device would provide more detailed, broad, and reliable results. In addition, as research has been undertaken to discuss the saccade detection algorithm, our algorithm could be also enhanced for more accurate saccade detection. Finally, sampling frequency of eye tracking device can be also an important factor as discussed in (43, 44). While higher sampling frequency costs more in terms of price and power consumption of eye tracking device, lower sampling frequency may lead us to misestimate the saccade detection. It would be important to find the appropriate sampling frequency, considering the proper balance of cost and reliability of a device.

#### Measurement Parameters

As discussed in the previous sections, the time stamp data recorded with the present version of SRanipal SDK (version 1.1.0.1) needs to be improved. In addition to the time stamp, the following parameters have not been supported yet, as far as we have confirmed with HTC. The future version of SRanipal SDK would implement further update to validate these parameters.

- int timestamp
- bool convergence_distance_validity (combined eye data)
- float convergence_distance_mm (combined eye data)
- float eye_squeeze (eye expression data)
- float eye_frown (eye expression data)

Finally, we were not able to calculate the peak velocity of saccadic eye movement when we used the normalized pupil position data. Nonetheless, if a conversion mapping between normalized screen coordinate and degrees is available, we could also calculate the peak velocity from the pupil position data.

#### Evaluation of Ocular Dominance

Ocular dominance is one of the important factors that could affect the results of saccadic eye movement. A previous study observed that eye dominance influenced saccade amplitude as the participants with strong ocular dominance reached more accurate saccades toward the target in the hemifield opposite to the side of dominant eye than in the same side (45). While our investigation has shown that both gaze direction and pupil position data can be used to evaluate latency and error rate of saccadic eye movement, ocular dominance as well as peak velocity of saccades could be evaluated with only gaze direction data. As discussed in the previous section, we observed visually the difference in the range of eye movement between left and right eyes in the normalized pupil position data, whereas the clear visual difference was not seen in the gaze direction data (see **Figure 5**). The difference seen in the pupil position data could not be due to the factor of ocular dominance because we should have seen the similar difference in the gaze direction data if eye dominance caused the difference. Therefore, we suppose that gaze direction data would be a proper data set when ocular dominance is inspected.

### Potential of HMD-VR Technology

While we designed a simple VR environment to simulate a situation of conventional 2D graphic display, the VR headset has a unique and novel potential in providing immersive VR environments. A previous review suggested the benefits of VR-based measures in its sensitivity of detecting cognitive impairments (21). Another study also emphasized the importance of taking advantage of immersive VR environments for the assessment of AD (46). Assessment of saccadic eye movement with various levels of immersion in VR environments could lead us to new research findings. Moreover, while we target at using the VR headset for the saccadic eye movement of neurodegenerative disorders, the device could become an alternative assessment tool in different fields. For example, if the VR headset is combined with a postural assessment device, we could stimulate postural sway with immersive VR environments and achieve a cost-efficient and portable assessment system to measure in a small space, instead of being restricted to dedicated laboratories with non-transportable systems for the balance assessment (47). Future studies integrating the VR device bear potential for assessments of diverse research fields.

## Conclusion

In our growing ageing society globally, neurodegenerative disorders are becoming a more relevant problem. Assessment of saccadic eye movement in those suffering from the disorders could be a promising way to diagnose them in a simple, time-efficient, and low-cost manner. Along with the advanced technologies of video-based eye tracking and VR, HTC launched a VR headset, VIVE Pro Eye, consisting of an infrared-based eye tracker and HMD. The purpose of this study was therefore to evaluate whether the VIVE Pro Eye could be used as an assessment tool of saccadic eye movement. We measured saccadic eye movement of seven healthy young adults using the product, following an internationally proposed standard protocol of saccade measurement. Our investigation results suggest that VIVE Pro Eye can function as an assessment device of saccadic eye movement and record the data necessary to calculate the oculo-metrics of latency, peak velocity, and error rate of saccades, and pupillary response. We also found technical limitations related to the eye tracking, especially about time-related measurement parameters. Further improvements in the provided SDK and the measurement protocol including saccade detection algorithm, and more measurements with diverse age-groups and those with different health conditions are necessary to enhance the whole assessment system of saccadic eye movement.

## Data Availability Statement

All data can be shared upon reasonable request to the first author. Programming background information is available on GitHub: https://github.com/MotorControlLearning.

## Ethics Statement

The studies involving human participants were reviewed and approved by ETH Zurich Ethics Commission. The patients/participants provided their written informed consent to participate in this study.

## Author Contributions

Each of the authors has contributed to developing the research concept and experimental designs. YI and AF developed the assessment system and performed the measurement. All authors conducted the first analysis and interpretation of the data. In addition, all authors contributed in drafting and revising the article to bring it to its current state. All authors contributed to the article and approved the submitted version.

## Conflict of Interest

The authors declare that the research was conducted in the absence of any commercial or financial relationships that could be construed as a potential conflict of interest.

## References

[1] United Nations World population ageing 2019. Tech. rep., New York, the USA: United Nations (2019).

[2] World Health Organization Decade of healthy ageing 2020-2030. Tech. rep., Geneva, Switzerland: World Health Organisation (2020).

[3] World Health Organization 10 priorities for a decade of action on healthy ageing. Tech. rep., Geneva, Switzerland: World Health Organisation (2017a).

[4] World Health Organization Global strategy and action plan on ageing and health. Tech. rep., Geneva, Switzerland: World Health Organisation (2017b).

[5] Heemels M-T Neurodegenerative diseases. Nature (2016) 539(7628):179–9. 10.1038/539179a 27830810

[6] Feigin VL Abajobir AA Abate KH Abd-Allah F Abdulle AM Abera SF Global, regional, and national burden of neurological disorders during 1990-2015: a systematic analysis for the global burden of disease study 2015. Lancet Neurol (2017) 16:877–97. 10.1016/s1474-4422(17)30299-5 PMC5641502 28931491

[7] Gorges M Pinkhardt EH Kassubek J Alterations of eye movement control in neurodegenerative movement disorders. J Ophthalmol (2014) 2014:11. 10.1155/2014/658243 PMC4052189 24955249

[8] MacAskill MR Anderson TJ Eye movements in neurodegenerative diseases. Curr Opin Neurol (2016) 29:61–8. 10.1097/wco.0000000000000274 26641817

[9] Marandi RZ Gazerani P Aging and eye tracking: in the quest for objective biomarkers. Future Neurol (2019) 14:22. 10.2217/fnl-2019-0012

[10] Molitor RJ Ko PC Ally BA Eye movements in alzheimer’s disease. Journal of. Alzheimers Dis (2015) 44:1–12. 10.3233/jad-141173 PMC5332166 25182738

[11] Wilcockson TDW Mardanbegi D Xia BQ Taylor S Sawyer P Gellersen HW Abnormalities of saccadic eye movements in dementia due to alzheimer’s disease and mild cognitive impairment. Aging-Us (2019) 11:5389–98. 10.18632/aging.102118 PMC6710064 31375642

[12] Goffart L Saccadic Eye Movements. Academic Press: Oxford (2009). p. 437–44. 10.1016/B978-008045046-9.01101-3

[13] Ramat S Leigh RJ Zee DS Optican LM What clinical disorders tell 751 us about the neural control of saccadic eye movements. Brain (2007) 130:10–35. 10.1093/brain/awl309 17121745

[14] Terao Y Fukuda H Hikosaka O What do eye movements tell us about patients with neurological disorders? - an introduction to saccade recording in the clinical setting. Proc Jpn Acad Ser B-Physical Biol Sci (2017) 93:772–801. 10.2183/pjab.93.049 PMC5790757 29225306

[15] Termsarasab P Thammongkolchai T Rucker JC Frucht SJ The diagnostic value of saccades in movement disorder patients: a practical guide and review. J Clin Mov Disord (2015) 2:14. 10.1186/s40734-015-0025-4 26788350 PMC4710978

[16] Liu Y Tan WJ Chen C Liu CY Yang JZ Zhang YC A review of the application of virtual reality technology in the diagnosis and treatment of cognitive impairment. Front Aging Neurosci (2019) 11:280. 10.3389/fnagi.2019.00280 31680934 PMC6813180

[17] Tarnanas I Tsolakis A Tsolaki M Assessing Virtual Reality Environments as Cognitive Stimulation Method for Patients with MCI. In: Brooks A Brahnam S Jain L, editors. Technologies of Inclusive Well-Being. Studies in Computational Intelligence, vol 536. Berlin, Heidelberg: Springer (2014). 10.1007/978-3-642-45432-5_4

[18] Benyoucef Y Lesport P Chassagneux A The emergent role of virtual reality in the treatment of neuropsychiatric disease. Front Neurosci (2017) 11:491. 10.3389/fnins.2017.00491 28928630 PMC5591848

[19] Moreno A Wall KJ Thangavelu K Craven L Ward E Dissanayaka NN A systematic review of the use of virtual reality and its effects on cognition in individuals with neurocognitive disorders. Alzheimer’s Dementia (New York N Y) (2019) 5:834–50. 10.1016/j.trci.2019.09.016 PMC6881602 31799368

[20] Dockx K Bekkers EMJ Van den Bergh V Ginis P Rochester L Hausdorff JM Virtual reality for rehabilitation in parkinson’s disease. Cochrane Database Syst Rev (2016) 12(12):CD010760. 10.1002/14651858.CD010760.pub2 28000926 PMC6463967

[21] Negut A Matu SA Sava FA David D Virtual reality measures in neuropsychological assessment: a meta-analytic review. Clin Neuropsychol (2016) 30:165–84. 10.1080/13854046.2016.1144793 26923937

[22] Muratore M Tuena C Pedroli E Cipresso P Riva G Virtual reality as a possible tool for the assessment of self-awareness. Front Behav Neurosci (2019) 13:62. 10.3389/fnbeh.2019.00062 31019454 PMC6458281

[23] Nolin P Banville F Cloutier J Allain P Virtual Reality as a New Approach to Assess Cognitive Decline in the Elderly, vol. 2 of 2013. Rome, Italy: Mediterranean Center of Social and Educational Research (2013).

[24] Antoniades C Ettinger U Gaymard B Gilchrist I Kristjansson A Kennard C An internationally standardised antisaccade protocol. Vision Res (2013) 84:1–5. 10.1016/j.visres.2013.02.007 23474300

[25] Hutton SB Ettinger U The antisaccade task as a research tool in psychopathology: A critical review. Psychophysiology (2006) 43:302–13. 10.1111/j.1469-8986.2006.00403.x 16805870

[26] Bijvank JAN Petzold A Balk LJ Tan HS Uitdehaag BMJ Theodorou M A standardized protocol for quantification of saccadic eye movements: Demons. PLoS One (2018) 13:19. 10.1371/journal.pone.0200695 PMC6047815 30011322

[27] Larsson L Nystrom M Stridh M Detection of saccades and postsaccadic oscillations in the presence of smooth pursuit. IEEE Trans Biomed Eng (2013) 60:2484–93. 10.1109/tbme.2013.2258918 23625350

[28] Hopf S Liesenfeld M Schmidtmann I Ashayer S Pitz S Age dependent normative data of vertical and horizontal reflexive saccades. PLoS One (2018) 13:13. 10.1371/journal.pone.0204008 PMC6143243 30226877

[29] Magnusdottir BB Faiola E Harms C Sigurdsson E Ettinger U Haraldsson HM Cognitive measures and performance on the antisaccade eye movement task. J Cognit (2019) 2:3. 10.5334/joc.52 31517223 PMC6634605

[30] Andersson R Larsson L Holmqvist K Stridh M Nystrom M One algorithm to rule them all? an evaluation and discussion of ten eye movement event-detection algorithms. Behav Res Methods (2017) 49:616–37. 10.3758/s13428-016-0738-9 27193160

[31] Coe BC Munoz DP Mechanisms of saccade suppression revealed in the anti-saccade task. Philos Trans R Soc B-Biol Sci (2017) 372:10. 10.1098/rstb.2016.0192 PMC5332851 28242726

[32] Seferlis F Chimona TS Papadakis CE Bizakis J Triaridis S Skoulakis C Age related changes in ocular motor testing in healthy subjects. J Vestib Res-Equilib Orientation (2015) 25:57–66. 10.3233/ves-150548 26410670

[33] Bargary G Bosten JM Goodbourn PT Lawrance-Owen AJ Hogg RE Mollon JD Individual differences in human eye movements: An oculomotor signature? Vision Res (2017) 141:157–69. 10.1016/j.visres.2017.03.001 28373058

[34] Munoz DP Broughton JR Goldring JE Armstrong IT Age-related performance of human subjects on saccadic eye movement tasks. Exp Brain Res (1998) 121:391–400. 10.1007/s002210050473 9746145

[35] Klein C Foerster F Hartnegg K Fischer B Lifespan development of pro- and anti saccades: Multiple regression models for point estimates. Dev Brain Res (2005) 160:113–23. 10.1016/j.devbrainres.2005.06.011 16266754

[36] Bradley JC Bentley KC Mughal A II Bodhireddy H Young RSL Brown SM The effect of gender and iris color on the dark-adapted pupil diameter. J Ocular Pharmacol Ther (2010) 26:335–40. 10.1089/jop.2010.0061 20698797

[37] Tekin K Sekeroglu MA Kiziltoprak H Doguizi S Inanc M Yilmazbas P Static and dynamic pupillometry data of healthy individuals. Clin Exp Optom (2018) 101:659–65. 10.1111/cxo.12659 29356077

[38] Rickmann A Waizel M Kazerounian S Szurman P Wilhelm H Boden KT Digital pupillometry in normal subjects. Neuro-Ophthalmology (2017) 41:12–8. 10.1080/01658107.2016.1226345 PMC5278786 28228832

[39] Murray NP Hunfalvay M Bolte T The reliability, validity, and normative data of interpupillary distance and pupil diameter using eye-tracking technology. Trans Vision Sci Technol (2017) 6:12. 10.1167/tvst.6.4.2 PMC5497600 28685104

[40] Andersson R Nystrom M Holmqvist K Sampling frequency and eye-tracking measures: how speed affects durations, latencies, and more. J Eye Mov Res (2010) 3:12. 10.16910/jemr.3.3.6

[41] Tobii Technology Timing Guide for Tobii Eye Trackers and Eye Tracking Software. Tech. rep., Danderyd, Sweden: Tobii Technology (2010).

[42] Appel L Appel E Bogler O Wiseman M Cohen L Ein N Older adults with cognitive and/or physical impairments can benefit from immersive virtual reality experiences: A feasibility study. Front Med (2020) 6(329). 10.3389/fmed.2019.00329 PMC6974513 32010701

[43] Leube A Rifai K Sampling rate influences saccade detection in mobile eye tracking of a reading task. J Eye Mov Res (2017) 10:11. 10.16910/jemr.10.3.3 PMC7141092 33828659

[44] Wierts R Janssen MJA Kingma H Measuring saccade peak velocity using a low frequency sampling rate of 50 hz. IEEE Trans Biomed Eng (2008) 55:2840–2. 10.1109/tbme.2008.925290 19126467

[45] Tagu J Dore-Mazars K Lemoine-Lardennois C Vergilino-Perez D How eye dominance strength modulates the influence of a distractor on saccade accuracy. Invest Ophthalmol Visual Sci (2016) 57:534–43. 10.1167/iovs.15-18428 26873513

[46] Garcia-Betances R II Waldmeyer MTA Fico G Cabrera-Umpierrez MF A succinct overview of virtual reality technology use in alzheimer’s disease. Front Aging Neurosci (2015) 7:80. 10.3389/fnagi.2015.00080 26029101 PMC4428215

[47] Harro CC Garascia C Reliability and validity of computerized force platform measures of balance function in healthy older adults. J Geriatr Phys Ther (2019) 42:E57–66. 10.1519/jpt.0000000000000175 29324510

